# Circulating Vitreous microRNA as Possible Biomarker in High Myopic Eyes with Macular Hole

**DOI:** 10.3390/ijms23073647

**Published:** 2022-03-26

**Authors:** Yoshimasa Ando, Hiroshi Keino, Makoto Inoue, Kazunari Hirota, Hiroyuki Takahashi, Kimihiko Sano, Takashi Koto, Tomohito Sato, Masaru Takeuchi, Akito Hirakata

**Affiliations:** 1 Department of Ophthalmology, Kyorin University School of Medicine, 6-20-2 Shinkawa, Mitaka, Tokyo 181-8611, Japan; yando@ks.kyorin-u.ac.jp (Y.A.); inoue-eye@ks.kyorin-u.ac.jp (M.I.); hirota@eye-center.org (K.H.); t.hiroyuki.oph@tmd.ac.jp (H.T.); sano@eye-center.org (K.S.); koto@ks.kyorin-u.ac.jp (T.K.); hirakata@ks.kyorin-u.ac.jp (A.H.); 2 Department of Ophthalmology and Visual Science, Tokyo Medical and Dental University, 1-5-45, Yushima, Bunkyo-ku, Tokyo 113-8519, Japan; 3 Department of Ophthalmology, National Defense Medical College, 3-2, Namiki, Tokorozawa 359-8513, Japan; dr21043@ndmc.ac.jp (T.S.); masatake@ndmc.ac.jp (M.T.)

**Keywords:** microRNA, axial length, high myopia, ocular inflammation, cytokines

## Abstract

High myopia is a major cause of irreversible visual impairment globally. In the present study, we investigated the microRNA (miRNA) profile in the vitreous of macular hole (MH) and high myopic MH. We performed miRNA analysis using TaqMan^®^ Low Density Arrays (Thermo Fisher Scientific, Waltham, MA, USA) to investigate the circulating vitreous miRNA profile from patients with MH (axial length < 26.5 mm, n = 11) and high myopic MH (axial length ≥ 26.5 mm, n = 11) who underwent pars plana vitrectomy. The vitreous inflammatory cytokine signature was examined in high myopic MH eyes using a multiplex assay. A miRNA-Array analysis revealed that let-7c was significantly up-regulated and miR-200a was significantly down-regulated in high myopic MH eyes compared to those in MH eyes. The bioinformatics analysis for up-regulated miRNA targeted gene identified 23 pathways including mitogen-activated protein kinase (MAPK) and several inflammatory signaling pathways, whereas the bioinformatics analysis for down-regulated miRNA targeted genes showed 32 enriched pathways including phosphoinositide 3-kinase/protein kinase B (PI3K/AKT). The levels of inflammatory cytokines including IP-10, IFN-γ, and MCP-1 were significantly higher in the vitreous of high myopic MH eyes. These results suggest that specific miRNAs expressed in the vitreous may be associated with the pathological condition of high myopic MH and the above mentioned miRNAs may contribute to the development of inflammatory status in the vitreous of high myopic eyes.

## 1. Introduction

High myopia is a major cause of irreversible visual impairment globally, especially in East Asian areas, with an increasingly high prevalence in the past few decades [1,2]. High myopia increases the risk of development of sight-threatening conditions such as retinal detachment, including rhegmatogenous retinal detachment or myopic macular traction, myopic choroidal neovascularization, and chorioretinal atrophy. Although both genetic and environmental factors have been considered to contribute to the development of these pathological processes, the cause of high myopia is not fully understood [3,4,5].

Pathologic myopia is associated with visual loss due to the degenerative process in the posterior segment produced by increased axial length and stretching of the eye wall [6,7]. Thus far, previous studies using the form-deprived myopia (FDM) animal model have demonstrated that transforming growth factor-β (TGF-β), metalloproteinase 2 (MMP2), and tissue inhibitor of metalloproteinase-2 (TIMP-2) in sclera are associated with the development of myopia [8,9]. In addition, serious ocular pathologies including macular hole (MH), macular traction syndrome, and retinoschisis are associated with posterior vitreous detachment (PVD), and PVD-related retinal pathologies occur more frequently in patients with high myopia than in patients without high myopia [10,11,12,13,14,15]. Recent studies using optical coherence tomography (OCT) have demonstrated a high frequency of vitreoretinal abnormalities in high myopic eyes, suggesting that the abnormality of interface between retina and vitreous may contribute to the high frequency of PVD-related retinal pathologies in high myopic eyes [16,17]. However, the studies regarding molecular mechanism of MH formation in highly myopic eyes using human vitreous samples have been limited [18,19].

MicroRNAs (miRNAs), small noncoding RNAs, regulate gene expression through posttranscriptional mRNAs degradation or by preventing translation of target genes [20]. miRNAs contribute to crucial cellular processes, and dysregulation of miRNAs is involved in the development of various disorders [21,22]. It has been reported that miRNAs are present in the vitreous and that altered expression of miRNAs is observed in the vitreous from different vitreoretinal diseases, such as MH, epiretinal membrane (ERM), rhegmatogenous retinal detachment, and proliferative diabetic retinopathy [23,24,25,26,27]. Yet no study to date has evaluated the changes in the profile of vitreous miRNA in high myopic eyes. Accordingly, it is important to characterize the miRNA expression profile of vitreous in high myopic MH eyes for understanding the molecular pathological process of MH formation and postoperative healing process as well as for the development of prognostic biomarker and prophylactic therapy for MH in high myopic eyes.

In the current study, we investigated the miRNA profile in the vitreous of MH and high myopic MH using real-time quantitative polymerase chain reaction (PCR)-based TaqMan microRNA arrays, and we determined the correlation between the expression level of each miRNA in the vitreous and axial length. Furthermore, since recent reports have demonstrated that microRNAs have pathogenic, therapeutic, and diagnostic roles in inflammatory disorders [28,29], we evaluated the vitreous inflammatory cytokine signature in high myopic MH eyes.

## 2. Results

### 2.1. Demographics

Demographic characteristics of patients with MH and high myopic patients with MH are summarized in Table 1. The MH group had 11 patients (5 men and 6 women) and the high myopic MH group had 11 patients (5 men and 6 women) (*p* = 1.00). The mean age (±SD) of the patients in the MH group was 67.5 ± 8.2, ranging from 48 to 77, and the mean age (±SD) of the patients in the high myopic MH group was 59.5 ± 14.5, ranging from 31 to 77 (*p* = 0.17). The MH group had 10 phakic eyes and 1 pseudophakic eye and the high myopic MH group had 8 phakic eyes and 3 pseudophakic eyes preoperatively (*p* = 0.59). The intervals between cataract surgery and pars plana vitrectomy (PPV) were 60 months in MH group and 7 months, 30 months, and 48 months in the High MH group. The mean preoperative axial length in the MH eyes was 23.8 ± 1.2 mm, ranging from 22.1 to 25.5 mm; the mean preoperative axial length in high myopic MH eyes was 28.3 ± 1.0 mm, ranging from 27.4 to 30.7 mm (*p* < 0.001). The preoperative best corrected visual acuity (BCVA) in MH eyes was 0.70 ± 0.33, ranging from 0.30 to 1.40; the preoperative BCVA in high myopic MH eyes was 0.40 ± 0.23, ranging from 0.097 to 0.82 (*p* = 0.02). Both the MH eyes (11 eyes) and high myopic MH eyes (11 eyes) in the current study showed idiopathic MH. The number of eyes in each MH stage was 1 (stage 1), 3 (stage 2), 2 (stage 3), and 5 (stage 4) in MH eyes, and the number of eyes in each MH stage was 0 (stage 1), 3 (stage 2), 4 (stage 3), and 4 (stage 4) in high myopic MH eyes according to the Gass classifications [30].

### 2.2. Differential Expression of microRNA between MH Eyes and High Myopic MH Eyes

**Figure 1:**
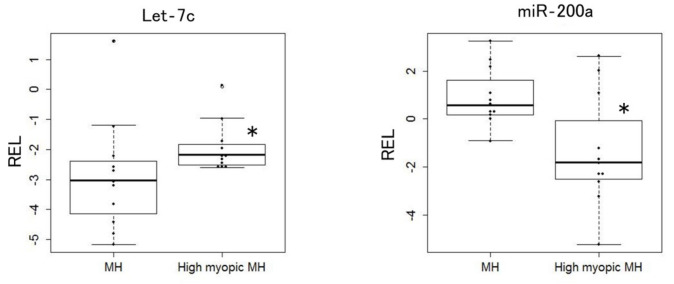
Differentially expressed miRNA in the vitreous of MH eyes and high myopic MH eyes. Box plots of vitreous levels of up-regulated miRNA (let-7c) in high myopic eyes, whereas down-regulated miRNA (miR-200a) in high myopia. The lines inside the boxes denote the median. * *p* < 0.05, Wilcoxon rank sum test was used for comparison between 2 groups. The amount of target miRNAs was normalized relative to the amount of (median Ct + U6)/2 and the data was presented as relative expression levels (RELs): ΔCt = Ct [(median Ct + U6)/2] − Ct [target miRNA]. MH: macular hole.

We employed Low Density Arrays (TLDAs) MicroRNA Array to compare the expression level of 381 microRNAs in the vitreous between MH eyes and high myopic MH eyes. We found the expression level of 2 miRNAs in the vitreous (let-7c and miR-200a) significantly altered between MH eyes and high myopic MH eyes. As shown in Figure 1, the expression level of let-7c (*p* = 0.034) was significantly higher in the vitreous of high myopic MH eyes compared with that of the vitreous of MH eyes, whereas the expression level of miR-200a (*p* = 0.023) was significantly lower in the vitreous of high myopic MH eyes compared with that of the vitreous of MH eyes. Although we investigated the correlation coefficients between the expression level of the 2 differentially expressed miRNAs (let-7c and miR-200a) and axial length of a total of 22 eyes (11 MH eyes and 11 high myopic MH eyes), no significant correlation was observed between the expression level of the 2 miRNAs (let-7c and miR-200a) and axial length.

### 2.3. Enrichment Analysis of Target Genes of Differentially Expressed miRNAs

**Figure 2:**
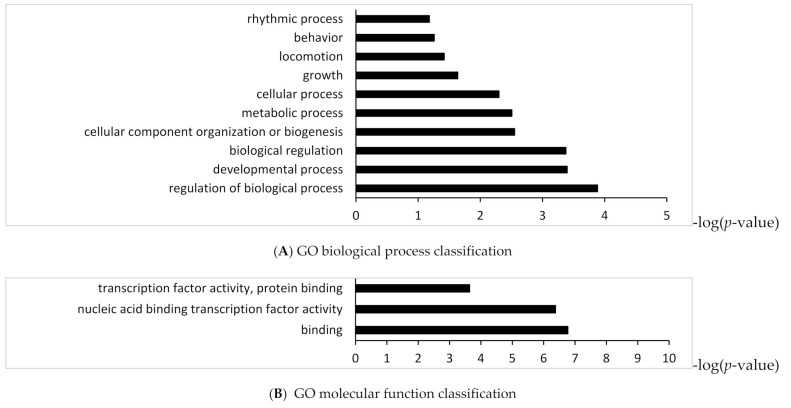

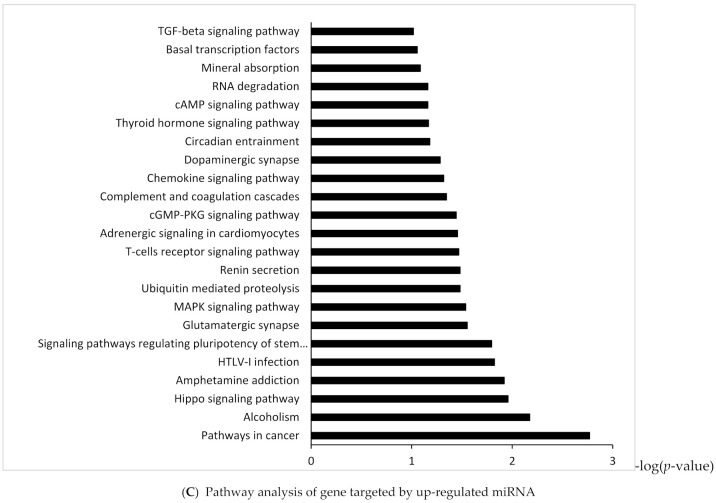
Gene Ontology and KEGG enrichment analysis of target genes of up-regulated miRNA in vitreous of high myopic MH eyes.

**Figure 3:**
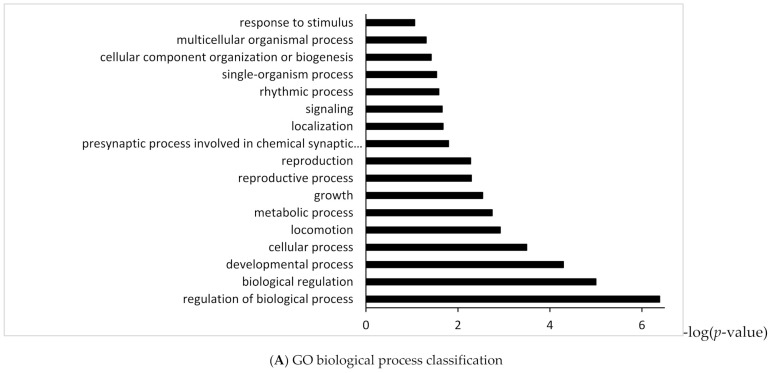

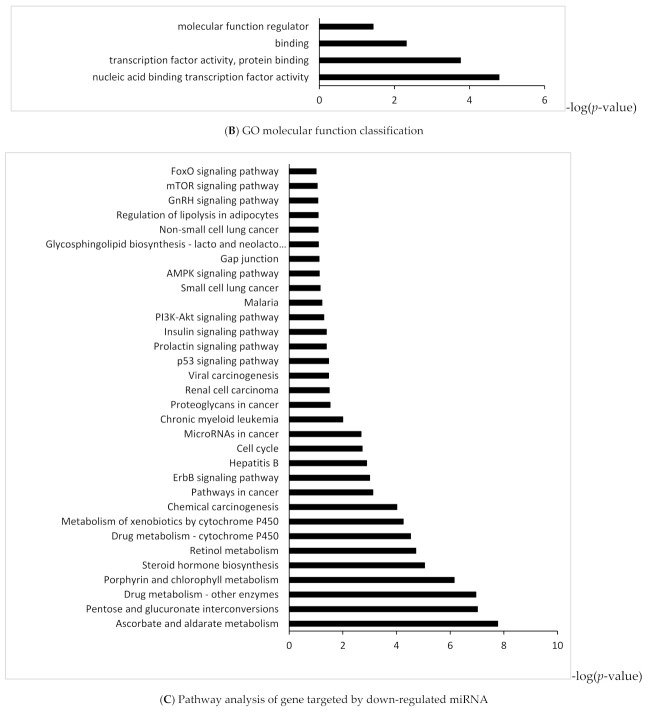
Gene Ontology and KEGG enrichment analysis of target genes of down-regulated miRNA in vitreous of high myopic MH eyes.

To identify the functional categories and pathway which were targeted by up-regulated or down-regulated miRNAs in high myopic MH eyes compared with MH eyes, we performed enrichment analyses based on Gene Ontology and KEGG (Kyoto Encyclopedia of Genes and Genomics) with the DAVID (Database for Annotation Visualization and Integrated Discovery). Gene ontology enrichment analysis for up-regulated miRNA targeted genes revealed 10 biological processes and 3 molecular functions (Figure 2A,B). KEGG pathway analysis for up-regulated miRNA targeted genes identified 23 pathways including pathways in cancer, signaling pathway regulating pluripotency of stem cells, and mitogen-activated protein kinase (MAPK), T-cell receptor signaling pathway, and chemokine signaling pathway in the vitreous of high myopic MH eyes (Figure 2C). Gene Ontology enrichment analysis for down-regulated miRNA targeted genes revealed 17 biological processes and 4 molecular functions (Figure 3A,B). KEGG pathway analysis for down-regulated miRNA targeted genes showed 32 enriched pathways including phosphoinositide 3-kinase/protein kinase B (PI3K/AKT) and regulating growth and reprogramming metabolism pathway including AMP-activated protein kinase (AMPK) signaling pathway, p53 signaling pathway and mTOR signaling pathway in vitreous of high myopic MH eyes (Figure 3C).

### 2.4. Comparison of the Levels of Inflammatory Cytokines in the Vitreous between MH Eyes and High Myopic MH Eyes

**Figure 4:**
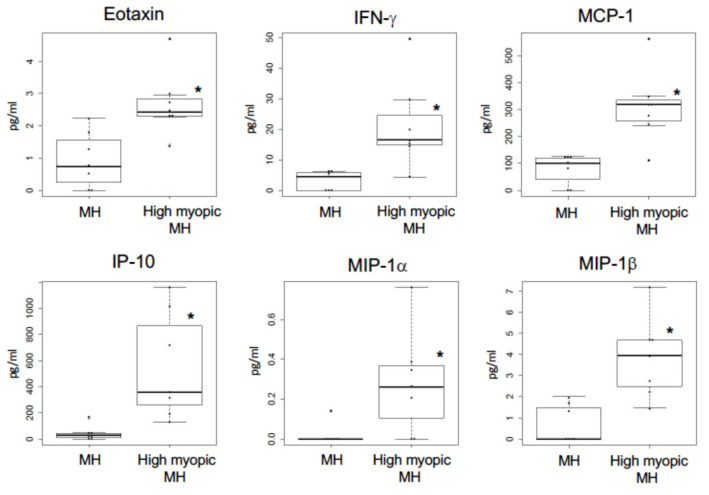
Comparison of inflammatory cytokines in the vitreous between MH eyes and high myopic MH eyes. Box plots of vitreous levels of up-regulated inflammatory cytokines in high myopic eyes. The lines inside the boxes denote the median. * *p* < 0.05, Wilcoxon rank sum test was used for comparison between 2 groups.

As shown in Figure 2C and Figure 3C, since KEGG pathway analysis for significantly altered miRNAs in the vitreous between MH eyes and high myopic MH eyes revealed several inflammatory pathways, including MAPK signaling pathway, T-cell receptor signaling pathway, and chemokine signaling pathway, we investigated the levels of 27 inflammatory cytokines in the vitreous between MH eyes and high myopic MH eyes. As shown in Figure 4, the levels of eotaxin, interferon-γ (IFN-γ), monocyte chemotactic protein-1 (MCP-1), interferon-inducible protein 10 (IP-10), macrophage inflammatory protein-1α (MIP-1α), and MIP-1β were significantly elevated in the vitreous of high myopic MH eyes (eotaxin; *p* = 0.016, IFN-γ; *p* = 0.010, MCP-1; *p* = 0.007, IP-10; *p* = 0.003, MIP-1α; *p* = 0.024, MIP-1β; *p* = 0.004,). There was no significant difference between either group regarding the levels of other cytokines.

### 2.5. Correlation between Inflammatory Cytokine Levels in the Vitreous and Axial Length

We analyzed correlation coefficients between cytokine levels and axial length of a total of 14 eyes (7 MH eyes and 7 high myopic MH eyes), and we found that 7 cytokines (interleukin (IL)-8, eotaxin, IFN-γ, MCP-1, IP-10, MIP-1α, MIP-1β) revealed significant positive correlation to axial length (Table 2).

## 3. Discussion

Using real time PCR-based array profiling, we identified circulating miRNAs that were altered specifically in the vitreous of high myopic MH eyes compared with that of MH eyes. We observed that let-7c was significantly elevated in the vitreous of high myopic MH eyes compared with that of MH eyes, whereas miR-200a was significantly decreased in the vitreous of high myopic MH eyes. Functional pathway analysis of the 2 dysregulated miRNA identified several signaling pathways including pathways in cancer, signaling pathway regulating pluripotency of stem cells, and mitogen-activated protein kinase (MAPK)/phosphoinositide 3-kinase/protein kinase B (PI3K/AKT) and AMP-activated protein kinase (AMPK) signaling pathway in the vitreous of high myopic MH eyes.

The present study demonstrated that the expression of let-7c was significantly elevated in high myopic vitreous. Let-7c has been identified to regulate TGF-β1 induced fibrosis [31,32]. Several reports have shown the association between let-7 family, including let-7c, and remodeling collagen in sclera [33,34]. Metlapally et al. revealed that the expression of let-7b and let-7c was significantly higher in fetal posterior sclera compared to that in adult eyes [34]. In addition, Akamine et al. have shown that let-7c increased in the vitreous of aging rats and let-7c was expressed in vitro by Müller glial cells and their extracellular vesicles [35]. Furthermore, the authors demonstrated that let-7c targeted hyaluronic acid synthases 2 (HAS2) mRNA, a synthesizing enzyme of hyaluronic acid [35]. Our group has recently shown that cellular components of glial cells were present on the surface of the internal limiting membrane (ILM) excised from highly myopic eyes with myopic traction maculopathy [36]. Although it remains unknown what cells secrete the let-7c in the vitreous, glial cells migrating on to the surface of ILM may contribute to production of let-7c in the vitreous of high myopic eyes.

In the present study, miR-200a was down-regulated in high myopic vitreous and KEGG pathway analysis demonstrated that PI3K/AKT signaling was a significantly targeted pathway by down-regulated miRNA (miR-200a). Recent studies have demonstrated that miR-200a inhibits proliferation of tumor cells by regulating the PI3K/AKT signaling pathway [37,38]. The PI3K-AKT signaling pathway has been associated axial eye growth via intravitreal insulin [39,40]. Li et al. indicated that the insulin receptor activation resulted in the phosphorylation of AKT and mammalian target of rapamycin (mTOR) in retinal pigments epithelial cell line (ARPE-19), leading to the secretion of pathological myopia related proteins such as insulin-like growth factor-1 (IGF-1) and matrix metalloproteinase-2 (MMP)-2, and this effect was suppressed by the PI3K inhibitor LY294002 [40]. Taken together, dysregulation of miR-200a in the vitreous may affect axial length progression via affecting the PI3K/AKT signaling pathway in high myopic eyes.

In the current study, KEGG pathway analysis for significantly altered miRNAs in the vitreous between MH eyes and high myopic MH eyes revealed several inflammatory pathways. Thus we investigated the level of inflammatory cytokines and chemokines in vitreous of MH eyes and high myopic MH eyes. We observed that eotaxin, IFN-γ, IP-10, MCP-1, MIP-1α, and MIP-1β in the vitreous was significantly higher in high myopic MH eyes than MH eyes. Furthermore, the levels of IL-8, eotaxin, IFN-γ, IP-10, MCP-1, MIP-1α, and MI-1β were significantly correlated with axial length. Yuan et al. have revealed that aqueous humor in high myopic eyes shows proinflammatory status [41]. Previous studies have reported that patients with acute zonal occult outer retinopathy (AZOOR) complex disorders proposed by Gass, a spectrum of chorioretinal disease, including multiple evanescent white dot syndrome (MEWDS), and punctate inner choroidopathy (PIC) as well as AZOOR, tend to be highly myopic [42,43,44,45]. Lin and colleagues have demonstrated that patients with inflammatory disorders, including type 1 diabetes mellitus, uveitis, and systemic lupus erythmatosus, tend to get myopia [46], suggesting that chronic intraocular inflammation in the anterior and posterior segment may be associated with myopia progression. In particular, the present study revealed that the levels of T-helper 1 (Th1) related cytokines, IFN-γ, and IP-10 in the vitreous was significantly higher in high myopic MH eyes, indicating that vitreous cytokine levels of high myopic MH eyes represent the Th1 phenotype. These findings are consistent with recent study by Wei et al. showing that the levels of IFN-γ, IP-10, MCP-1, eotaxin, and MIP-1 were significantly higher in the high-myopic MH group than in the MH group [47]. In the present study, KEGG pathway analysis identified several inflammatory pathways including MAPK signaling pathway, T-cell receptor signaling pathway, and chemokine signaling pathway, suggesting that inflammatory status including Th1-related cytokines may contribute to the pathological condition of high myopic MH eyes.

The present study demonstrated that the level of MCP-1 in the vitreous was significantly higher in the high myopic MH eyes compared to the MH eyes. MCP-1 plays an important role in the recruitment of monocytes [48]. It has been reported that MCP-1 is expressed from Müller glial cells and the level of MCP-1 in the vitreous was elevated after acute retinal detachment (RD) in mouse models [49,50]. Matsumoto and colleagues demonstrated that mechanical stimulation by RD-induced Müller glial cell swelling, resulting in the release of MCP-1 via activation of transient receptor potential vanilloid 4 in Müller glial cell swelling [51]. It is well known that excessive elongation of the eyeball in high myopic eyes leads to mechanical stretching of retinal tissue, including Müller cells, resulting in degenerative changes in the posterior segment of the eye [1]. It remains unknown what cells secrete MCP-1 in the vitreous in high myopic MH eyes, and further study is necessary to determine the association between stretched Müller glial cells by long-standing mechanical stress and the expression of MCP-1.

In the current study, only two miRNAs were identified as differentially expressed miRNAs between MH eyes and highly myopic MH eyes. These results may be due to the sampling site of the vitreous. Undiluted vitreous samples (0.5–2.0 mL) were aspirated from the mid-vitreous at the beginning of the surgery in the present study. Recently, our ultrastructural analysis has revealed the cellular proliferation of glial cells, RPE-like cells, and myofibroblast-like cells on the excised ILMs in highly myopic eyes with myopic traction maculopathy, and the cells that migrate onto the surface of the ILM synthesize new collagen [28]. These findings may indicate that vitreous samples close to the surface of the retina (vitreoretinal interface) may represent the more characteristic miRNA expression profile of highly myopic eyes compared to that of mid-vitreous samples.

In the present study, let-7c was significantly elevated in the vitreous of high myopic MH eyes compared with that of MH eyes, whereas miR-200a was significantly decreased in the vitreous of high myopic MH eyes. There is accumulating evidence that a number of miRNAs are involved in retinal neurogenesis and retinal diseases [52]. The let-7 family plays as key regulators of the early-to-late developmental transition in retinal progenitors and promote rod survival [53,54]. The miR-200b has regulatory roles on vascular endothelial growth factor-mediated pathologic changes in diabetic retinopathy [55]. Although it remains to be addressed that elevated expression of let-7c in the vitreous is due to the recovery of the ellipsoid zone in the macular area after MH surgery, the high expression of let-7c in the vitreous may be associated with early recuperation of the retinal outer segment in high myopic MH eyes.

The current study investigated the miRNA expression profile of vitreous in high myopic MH eyes and putative roles of differentially expressed miRNAs were determined using pathway analysis. Our results may provide basic information for understanding the molecular pathogenesis of MH and for the development of prognostic biomarkers and new therapeutic strategies, including prophylactic therapy for MH in high myopic eyes. Further functional investigations are required to figure out the regulatory roles of the 2 dysregulated miRNAs (let-7c and miR-200a) and how these miRNAs contribute to the development of MH in high myopic eyes. Further in vivo study using animal model or in vitro assay using cultured Müller glial cells or retinal pigment epithelial cells by up- or down-regulation of let-7c/miR-200a are needed to confirm these results.

We find it necessary to consider some limitations of our present study. First, our sample size was not large enough to draw definitive conclusions, and the samples were obtained from a single institution. In addition, the miRNA expression of high myopic eyes with MH may only represent the miRNA expression in a small proportion of all high myopic eyes. Further studies are required to confirm our findings with a larger number of cases. Second, preoperative lens status was different between MH eyes (10 phakic eyes and 1 pseudophakic eye) and high myopic eyes (8 phakic eyes and 3 pseudophakic eyes). Since previous reports have revealed that several microRNA levels are associated with lens opacification [56,57], preoperative lens status may have an influence on the miRNA expression profile in the aqueous humor and vitreous of highly myopic MH eyes. Furthermore, enrollment of high aged patients in high myopic MH eyes may affect on the circulating miRNA signature in vitreous. Further validation studies of differentially expressed miRNAs in high myopic MH eyes in large cohorts and independent studies is warranted.

## 4. Materials and Methods

### 4.1. Patients

This study was conducted in accordance with the tenets of the Declaration of Helsinki and approved by the Research Ethics Committee of the Kyorin University School of Medicine (ethics approval number 663) on 6 April 2016, and written informed consent was obtained from each patient. All patients attended the Kyorin Eye Center, Kyorin University Hospital (Tokyo, Japan). The diagnosis of MH was based on a full ophthalmic evaluation, including ophthalmoscopy by non-contact lens slit-lamp biomicroscopy, spectral domain optical coherence tomography (SD-OCT) (Spectralis OCT, Heidelberg Engineering, Heidelberg, Germany), swept-source OCT (SS-OCT) (DRI OCT-1, Topcon, Tokyo, Japan) before PPV. PPV was performed for all of the patients at the Kyorin Eye Center between September 2017 and May 2018. The axial length was measured using OA2000 (TOMEY, Nagoya, Japan). The BCVA and clinical demographics were assessed. BCVA was converted to the logMAR units for statistical analyses. An axial length ≥ 26.5 mm was defined as the eyes with high myopia. Eyes with previous vitreoretinal surgery, diabetic retinopathy, high blood pressure retinopathy, uveitis, retinal vein occlusion, trauma, and other retinal diseases were excluded. The miRNA assay was performed for vitreous samples from 11 patients with MH and 11 high myopic patients with MH and the intraocular cytokine and chemokine assay was performed for vitreous samples from 7 of 11 patients with MH and 7 of 11 high myopic patients with MH.

### 4.2. Surgical Procedures and Sample Collection

PPV was performed by five experienced vitreoretinal surgeons (H.T., K.S., T.K., K.S., and A.H.) at the Kyorin Eye Center. The surgical procedure was described previously [58]. Briefly, a 25-gauge vitrectomy system (Alcon Constellation^®^ Vision System, Alcon Laboratories, Fort Worth, TX, USA) with a cutting rate of 5000 cuts/min and aspiration pressure of 650 mmHg was used. The lens was extracted from patients if the eye was phakic followed by phacoemulsification with simultaneous implantation of an intraocular lens. Before starting PPV, a 25G trocar was inserted 3.5 mm posterior to the corneal limbus by a transconjunctival one-step incision. The vitreous (0.5–2.0 mL) was collected before the infusion, and it was immediately centrifuged at 400× *g* (1500 rpm) for 10 min to remove any cell debris. All samples were preserved at −80 °C until assayed.

### 4.3. Sample Preparation

The frozen vitreous was thawed on ice. Total RNA was extracted from the vitreous using the Qiagen miRNeasy^®^ Mini Kit (#217184, Qiagen, GmbH, Hilden, Germany) following the manufacturer’s protocol. For the microRNA assay, a 200 μL aliquot of the vitreous samples was used. Finally, the total RNA was eluted in 14 μL of RNase-free water. The concentration of RNA was analyzed by measuring a 2 µl aliquot on a NanoDrop ND-3300 Fluorospectrometer (Thermo Fisher Scientific, Waltham, MA, USA). The High Sensitivity RNA ScreenTape^®^ (Agilent Technologies, Santa Clara, CA, USA) was used to assess the RNA quality of our samples according to the manufacturer’s instructions.

### 4.4. Reversal Transcription and Quantitative PCR Using microRNA Array

Total RNA (22 µg/3 µL) was reverse transcribed using TaqMan™ microRNA Reverse Transcription Kit (#4366596, Applied Biosystems; Thermo Fisher Scientific, Waltham, MA, USA) and the Megaplex™ RT Primers HumanPool A v2.1 (#4399966, Applied Biosystems; Thermo Fisher Scientific, Waltham, MA, USA) to synthesize single-stranded cDNA. RT reaction had a final volume of 7.5 μL (3.0 μL total RNA and 4.5 μL RT reaction solution). The thermal cycling condition consisted of 40 cycles: 3-step cycling (16 °C for 2 min; 42 °C for 1 min; 50 °C for 1 s) followed by incubation at 85 °C for 5 min and 4 °C hold. The synthesized cDNA was preamplified to increase the quantity of the cDNA for gene expression analysis using TaqMan^®^ preAmp Master Mix (#4384266, Applied Biosystems; Thermo Fisher Scientific, Waltham, MA, USA) and Megaplex™ preAmp Primers Human Pool A v2.1 (#4399233, Applied Biosystems; Thermo Fisher Scientific, Waltham, MA, USA). Pre-amplification reaction had a final volume of 25 μL (2.5 μL RT product and 22.5 μL pre-amplification reaction mix solution). The reaction condition consisted of 95 °C for 10 min; 55 °C for 2 min; 72 °C for 2 min; 12 cycles, 2 step cycling (95 °C for 15 s; 60 °C for 4 min) followed by incubation at 99.9 °C for 10 min and 4 °C hold. Amplified products were loaded on TaqMan^®^ TLDAs TaqMan Human MicroRNA Array v2.0 A (#4398977, Applied Biosystems; Thermo Fisher Scientific, Waltham, MA, USA). PCR on TLDAs was performed on a QuantStudio 12K Flex Time PCR System (Applied Biosystems; Thermo Fisher Scientific, Waltham, MA, USA). The reaction condition consisted of 50 °C for 2 min; 95 °C for 10 min; 40 cycles, 2-step cycling (95 °C for 15 s; 60 °C for 1 min) followed by incubation at 4 °C hold. For normalization of these qPCR data using microRNA array, median Ct and U6 were selected as endogenous control miRNA [59]. The amount of target miRNAs was normalized relative to the amount of median Ct and U6. Relative expression levels (RELs) were presented as ΔCt = Ct [(median Ct + U6)/2 − Ct [target miRNA]. If miRNA Ct ≥ 36, the value was considered as undetermined and was set to Ct = 36 [60].

### 4.5. Measurement of Inflammatory Cytokines in the Vitreous

The concentrations of 27 cytokines, basic fibroblast growth factor (FGF), eotaxin, G-CSF (granulocyte colony-stimulating factor), GM-CSF (granulocyte-macrophage colony-stimulating factor), interferon-γ (IFN-γ), interleukin (IL)-1β, IL-1 receptor antagonist (IL-1ra), IL-2, IL-4, IL-5, IL-6, IL-7, IL-8, IL-9, IL-10, IL-12 (p70), IL-13, IL-15, IL-17, IFN Inducible Protein 10 (IP-10), monocyte chemotactic protein-1 (MCP-1), macrophage inflammatory protein-1α (MIP-1α), MIP-1β, platelet derived growth factor BB (PDGF-BB), regulated on activation, normal T-cell expressed and secreted (RANTES), tumor necrosis factor-α (TNF-α), vascular endothelial growth factor (VEGF), and vitreous humor samples were measured using a multiplex assay instrument (MAGPIX; Bio-Rad Laboratories, Hercules, CA, USA) according to the manufacturer’s instructions and previous report by Fukunaga and colleagues [61]. Levels of vitreous humor cytokines below detectable levels were given as 0 for statistical analysis.

### 4.6. Prediction of Target mRNAs

The putative mRNA targets for the differentially expressed miRNAs between MH eyes and high myopic MH eyes were retrieved with TargetScan database with the context score −0.2 or less.

### 4.7. Functional and Pathway Enrichment Analysis

Functional and pathway enrichment analyses for the differentially expressed miRNAs between MH eyes and high myopic MH eyes were performed based on Gene Ontology [62] and KEGG [63] with the DAVID (http://david.abcc.ncifcrf.gov/ (accessed on 22 February 2022)). Enriched Gene Ontology terms and KEGG pathways were selected using the Fisher’s exact test with a *p*-value threshold < 0.05.

### 4.8. Statistical Analysis

Statistical significance was evaluated by Wilcoxon rank sum test. Fisher’s exact test was applied for sex and lens status before PPV. We performed an a priori sample size calculation using the data of our previous study [24]. We found that for a statistical power of 0.80, the sample size for each group required in order to detect significant differences in levels of miRNAs was approximately 6. Therefore, we attempted to recruit around 6 cases each of MH eyes and high myopic MH eyes for this study. The data was analyzed by taking medians for each microRNA expression of all samples, not pooled samples. *p* values less than 0.05 were considered statistically significant. All analyses were performed using R software, version 3.5.1.3.

## 5. Conclusions

Our study demonstrated the altered expression of circulating miRNAs (let-7c and miR-200a) related to tissue remodeling, axial length elongation, myopia development, and inflammation in the vitreous of high myopic MH eyes, suggesting that specific miRNAs and inflammatory status in vitreous may be associated with the pathological condition of high myopic MH.

## Figures and Tables

**Table 1:** Characteristics and clinical findings. MH: macular hole; BCVA: best corrected visual acuity; LogMAR: logarithm of the minimum angle of resolution.

	MH (n = 11)	High Myopic MH (n = 11)	*p*
Sex (male, female)	5, 6	5, 6	1.00
Age (year, mean ± SD)	67.5 ± 8.2	59.5 ± 14.5	0.17
Lens status (phakia, pseudophakia)	10, 1	8, 3	0.59
Axial length (mm, mean ± SD)	23.8 ± 1.2	28.3 ± 1.0	<0.001
Preoperative BCVA (logMAR unit, mean ± SD)	0.70 ± 0.33	0.40 ± 0.23	0.02

**Table 2:** Spearman’s rank correlation rho and *p*-value between the levels of inflammatory cytokines in the vitreous and axial length.

	Rho	*p*-Value
IP-10	0.7701	0.001
IFN-γ	0.7160	0.004
MCP-1	0.6997	0.005
MIP-1α	0.6623	0.010
MIP-1β	0.6572	0.011
Eotaxin	0.6293	0.016
IL-8	0.5912	0.026
